# Text and picture integration during bridging information processing: A comparison of English and Chinese L1 and L2 speakers

**DOI:** 10.3758/s13421-025-01720-1

**Published:** 2025-05-20

**Authors:** Chenyi Zhang, Ianthi Tsimpli, Elaine Schmidt

**Affiliations:** 1 Department of Theoretical and Applied Linguistics, University of Cambridge, 9 West Road, Cambridge, CB3 9DP United Kingdom; 2 Cambridge Assessment, University of Cambridge, Shaftesbury Road, Cambridge, CB2 8EA United Kingdom

**Keywords:** Bridging information, Text and picture integration, Reading comprehension, Visual narratives, Verbal narratives, English, Chinese, Second language processing, Logographic scripts

## Abstract

Bridging information facilitates comprehension in both textual and pictorial stories, but its effects when both types of information are present remain unclear. This study examines how bridging information and individual differences influence comprehension across texts and pictures. Participants read four-segment stories under six conditions: 1) *picture-original* (pictorial stories), 2) *picture-to-text-switch* (pictorial stories with textual bridging information), 3) *picture-missing* (pictorial stories without bridging information), 4) *text-original* (textual stories), (5) text-to-picture-switch (textual stories with pictorial bridging information), and (6) text-missing (textual stories without bridging information). L1 and L2 Chinese and English speakers participated. Missing conditions led to longer comprehension times across all groups, confirming the facilitative role of bridging information. Crucially, the picture-to-text switch caused no disruption, and Chinese L1 speakers were unaffected by the text-to-picture switch, suggesting that bridging information can be processed during text and picture integration without extra cognitive resources. However, English L1 and L2 speakers, as well as Chinese L2 speakers, showed increased comprehension times in the text-to-picture switch condition, indicating greater processing difficulty for alphabetic and L2 texts. Robust effects of individual differences were also revealed.

## Introduction

### Text and picture integration

Reading comprehension, i.e., the act of deciphering written words and transforming them into meaningful mental representations, is a complex cognitive task that has interested psychologists who have studied discourse processing for a long time. Both L1 and L2 speakers of all age groups and languages have been widely examined, with typical or atypical development alike (e.g., Coloma et al., 2020; Garrod and Sanford, 1994; Kim et al., 2004; Linck et al., 2014; Long et al., 1990; Noble & Mccandliss, 2005; Ricketts, 2011; Van Dijk et al., 1983). However, such investigation has mainly focused on text reading only, whereas, in daily reading practices, individuals encounter not only textual information but also visual elements such as pictures, photos, symbols, emojis, emoticons, etc. For instance, readers of comics or manga naturally perceive images as integral components of a narrative, and they also need to take into consideration the accompanying texts or words to fully understand the stories.

However, the question of how picture stories are understood has long been neglected. Instead, the “Visual Ease Assumption” — the idea that visual stimuli are easier to comprehend than verbal stimuli — has been widely adopted (cf. Coderre, 2020). It is believed that pictures depict concrete objects and directly reflect their semantic representations, thus requiring fewer processing steps (Quill, 1997). Consequently, pictures are frequently employed in education for clinical population (McCleery et al., 2010), as well as second and foreign language learners (Kalaja et al., 2013).

Nonetheless, recent studies have demonstrated that while single pictures might be easier to understand than words, in complex settings, especially when narratives are formed, visual stimuli might not be straightforward, and the ability to understand visual narratives needs to be acquired (Coderre, 2020). For instance, Magliano et al. (2024) revealed that learners with lower literacy require greater cognitive resources to process and interpret images, and have difficulty in understanding pictorial stories. Consequently, the authors concluded that visual narratives comprehension demand specific interpretive skills. Further supporting this view, a meta-analysis by Coderre and Cohn (2024) demonstrated that proficiency in understanding visual narratives and the age at which these comprehension skills are acquired interact to affect the processing of ungrammatical visual narratives. To measure such proficiency, Cohn (2014a) developed the Visual Language Familiarity Index (VLFI). This questionnaire assesses participants’ engagement with visual narratives both presently and when growing up, which serves as an indicator of the proficiency in reading visual languages. Studies employing the VLFI have reported positive correlations between exposure to visual languages and performance in visual narrative processing, particularly in inferential processing (e.g., Cohn, 2013; Cohn and Kutas, 2015a; Cohn and Kutas, 2015b; Kirtley et al., 2018), although non-significant effects have also been demonstrated (e.g., Coderre et al., 2018; Hagmann and Cohn, 2016; Zhao et al., 2024). Further studies are still needed to offer a solid conclusion on this issue.

A following question is whether and how readers can integrate information across texts and pictures. While there is broad agreement that text and picture integration (TPI) can be achieved and even enhances comprehension and facilitates learning and generalization (e.g., Knoeferle et al., 2011; Mayer, 2005; Scharinger et al., 2020; for a review, see Renkl and Scheiter, 2017), it is still controversial whether readers build an integrated mental model or construct separate representations for textual and pictorial information.

A recent model that supports the notion of separate representations is the Integrated Model of Text-Picture Comprehension (ITPC) (Schnotz, 2014)^1^. According to this model, reading comprehension involves three hierarchical levels. First, written text and images are visually registered. This information is then transmitted to working memory (WM), where texts and images undergo separate routes: texts form graphemic lexical patterns, while images generate visuospatial patterns. Subsequently, different semantic processing is required to form two sets of mental representations: texts are parsed into propositional representations, whereas images are mapped onto mental models. Finally, these representations interact through model construction and inspection, eventually integrating into a coherent conceptual representation in long-term memory.

While the ITPC model offers a clear and detailed explanation of TPI, some critiques could be proposed. First, this model distinguishes between the propositional model (derived from texts) and the mental model (derived from pictures). However, propositional models are, by definition, representations of meaning and should be considered a subset or type of mental model (Kintsch, 1988). Such use of terms may thus cause confusion. Second, the model proposes that texts and pictures form separate mental models, which are later integrated through comparison. However, converging evidence has suggested that text and picture processing may share a common mechanism, and shared brain networks for semantic processing across texts and pictures have been identified (e.g., Shinkareva et al., 2011; Van Doren et al., 2010). Therefore, It is reasonable to argue that information from texts and pictures can be integrated into a single mental representation.

One of the models proposing a single mental representation is the Multimodal Parallel Architecture (Cohn, 2016; Klomberg & Cohn, 2022). This model serves as an extension of Jackendoff (2023)’s Parallel Architecture to explain how different forms of communication, such as text, speech, gestures, and pictures, are integrated within a single cognitive model. According to this model and the related *visual language theory*, pictures are drawn using representational systems, namely visual languages, in a similar way to verbal language, employing schematic patterns and a structured grammar (Cohn, 2013). These forms of communication share common underlying structures, namely modality, meaning, and grammar, which interact within a unified mental framework. Texts and pictures are processed through the same mental architecture, allowing them to form a cohesive mental model.

This integration is, of course, not merely the sum of two separate models; it involves complex grammatical and semantic interactions that enable a deeper understanding of multimodal messages. Substructures across modalities interact with each other to achieve the integration. Just as spoken or written language follows grammatical rules, visual narratives are organized according to the “visual language” with a structured “narrative grammar”. These patterns govern how individual images are arranged, much like the way words and sentences combine to form coherent stories (Cohn, 2013). In multimodal contexts, comprehenders break the visual and verbal expressions down into individual parts. While these parts differ across modalities, they collectively contribute to a shared **Conceptual Structure** (Cohn, 2016), which serves as the foundation for multimodal comprehension. Such contributions from different modalities can be either balanced, where they carry equal weight, or imbalanced, where one modality dominates the interpretation; Nonetheless, only one integrated cognitive system is ultimately built.

To summarize, given the ongoing debate, it remains unclear whether readers construct a single mental model or separate models for different modalities during TPI. This question has significant implications for theories of multimodal reading comprehension and cognitive processing. Therefore, further research is highly needed. The current study aims to investigate how bridging information is processed during TPI and how this process is affected by writing systems and individual differences, providing empirical insights into the mechanisms underlying multimodal reading comprehension.

### Bridging information and inferences

Inherent to reading comprehension is that readers not only process the given information but also need to retrieve information that is missing from the discourse. In both textual and pictorial stories, events between panels or sentences are often omitted. To fill in such gaps, bridging inferences have to be generated (Kintsch, 1994; McNamara & Magliano, 2009), which connect information across different parts of the stories and establish local coherence (Leong et al., 2008; Long et al., 1996).

Extensive research has been done to investigate bridging inference generation and processing in textual reading comprehension (for a review, see Brown, 1998). In one of the earliest studies, Haviland and Clark (1974) compared the reading times of sentence pairs with or without bridging information. To be more specific, some sentence pairs offered explicit bridging information such as *“Last Christmas Eugene became absolutely smashed. This Christmas he got very drunk again”* (Haviland & Clark, 1974, p.517). In the other sentence pairs, such information is only implied with adverbs, e.g., *again, still, too,* or *either*. An example sentence pair is *“Last Christmas Eugene went to a lot of parties. This Christmas he got very drunk again.”* (Haviland & Clark, 1974, p.517). In such cases, bridging inferences have to be generated in order to retrieve the missing bridging information. As hypothesized, readers needed significantly more time to read the latter type of sentence pairs, indicating that the bridging structure facilitates reading comprehension, and bridging inference generation requires additional cognitive resources. Such extra efforts in inferential generation and processing have been further established in later studies (e.g., Blanc et al., 2011; O’Brien et al., 1998; Pulgram, 1951; Rapp and Kendeou, 2007).

Meanwhile, some studies have revealed significant individual differences in such processes. One of such individual differences is WM, i.e., the memory system with limited capacity responsible for temporary information storage and updating (cf. Baddeley, 2003; Baddeley and Hitch, 1974). Positive correlations between WM capacity and reading abilities, especially inference processing, have been widely reported (e.g., Linderholm and van den Broek, 2002; Pulgram, 1951; Yeari, 2017). For example, Rai et al. (2011) found that readers with lower WM capacity face challenges in generating inferences during reading comprehension. Similarly, Pulgram (1951) showed that lower WM capacity is associated with difficulties in inference revision.

In recent years, an increasing interest has been in comprehending pictorial stories. Cohn (2013) introduced the concept of narrative grammar to explain how readers derive meaning from sequential images, which work similarly to discourse-level syntax. Inspired by this theory, several studies have explored reading comprehension of visual narratives, and comparable results between pictorial story comprehension and textual passage understanding have been reported, both within (e.g., Laubrock et al., 2018) and across panels (e.g., Foulsham et al., 2016; Foulsham and Kingstone, 2017).

Notably, just like how verbal WM influences reading comprehension, visuospatial WM has been suggested to play a role in the comprehension of visual narratives. For instance, Magliano et al. (2016) investigated the relative roles of visuospatial and verbal WM in inferential generation when reading visual narratives. These visual narratives either contained a bridging event (“present” condition”) or did not (“absent condition”). In the “absent” condition, a bridging inference had to be generated, and the participants needed more time to read the visual narratives. More importantly, this extra viewing time was modulated by both the verbal and visuospatial WM capacities of the readers, suggesting the significant effects of visuospatial WM on visual narrative processing. Such effect, however, is still controversial, as some studies failed to replicate such effects (e.g., Zhao et al., 2024).

More importantly, a few studies have directly investigated the processing of bridging information and the generation of bridging inferences when processing visual narratives. For example, Cohn and Kutas (2015a) conducted the first empirical study on bridging inference generation in pictorial stories using an event-related potential (ERP) methodology. Participants’ brain responses were recorded as they viewed pictorial stories with surprising endings. Three types of story conditions were created: in the first condition, no visual cues indicated that the ending would be surprising; in the second condition, the unexpected events leading to the surprising endings were explicitly depicted; and in the third condition, while the unexpected events themselves were not shown, visual cues provided hints. The results showed that the first condition elicited larger P600 effects compared to the two cued conditions, supporting the facilitative role of bridging information in visual narrative comprehension. Furthermore, when comparing the second and third conditions, the third condition elicited greater frontal negativity, suggesting increased cognitive demands for generating bridging inferences.

Additional evidence supporting these findings has been reported (e.g., Hutson et al., 2018; Magliano et al., 2017). For instance, through a behavioral experiment, Magliano et al. (2017) demonstrated that bridging inferences are crucial for connecting events and understanding the overall narrative when reading pictorial stories. Participants spent more time processing panels following omitted bridging events, indicating the effort to infer missing content. This effort was further evidenced by their performance in the recall task, where participants often misremembered inferred events as having been explicitly shown. These findings suggest that information from both text and pictures is processed through a shared mechanism, with bridging information playing a critical role in this integrative process.

However, only a few empirical studies have investigated TPI during bridging information processing. Substitution, i.e., instances where a unit of one modality is replaced by a structure from another modality (Cohn & Schilperoord, 2022), is the most commonly adopted manipulation method to examine this topic. Substitutions are frequently encountered in daily communication, literary work, and everyday life, exemplified by the iconic phrase “I  NY” created by Milton Glaser. For example, Manfredi et al. (2017) made the first attempt to address this issue. In this study, climactic events in pictorial stories were replaced with either onomatopoeic words that described the sounds of the events or descriptive words that explicitly described the events. The descriptive words could be either congruent or incongruent with the story context. The study analyzed the ERPs elicited by each condition. As expected, incongruent words elicited larger N400 effects compared to congruent words, indicating difficulties in semantic integration. When comparing onomatopoetic and descriptive words, the latter elicited greater late frontal positivities. To account for this result, the authors argued that onomatopoetic words are easier to integrate with the pictorial context, possibly due to their frequent use in comics. In contrast, less familiar descriptive texts posed greater challenges for integration. However, a major limitation of the study was the absence of a baseline condition involving only pictures, making it unclear whether TPI requires additional cognitive resources. Consequently, definitive conclusions about the cognitive demands of TPI could not be drawn.

Klomberg and Cohn (2022) conducted a series of controlled comparisons to examine the effects of replacing climactic events in visual narratives with five different types of inferential techniques, including onomatopoeic words. Not surprisingly, all inferential techniques led to longer processing time than the original panel, indicating the cognitive costs associated with inferential generation. Among the various inferential techniques, onomatopoeic words caused shorter reading times despite belonging to a different modality. A possible reason might be that onomatopoeic words contain fewer visual details than other pictorial counterparts, making them easier to process. Meanwhile, onomatopoeic words provided more explicit information and direct cues about the missing event, facilitating comprehension of the subsequent panels compared to implicit techniques like action stars or metaphoric symbols. The authors also investigated the effect of combining the onomatopoeia with other pictorial techniques, and found that story comprehension was not significantly affected. These findings suggest that substituted texts can be integrated with pictures during bridging information processing without distorting reading comprehension. Instead, visual complexity and explicitness might be more critical factors in determining the understanding of the story.

The only study to our knowledge that replaced a panel with a descriptive sentence is Huff et al. (2020). The authors adopted the viewing time paradigm and asked the participants to read pictorial stories comprising four panels, namely beginning state, bridging event, end state, and end state + 1. The bridging event panel was manipulated to achieve four conditions across three experiments: it was either present, absent, replaced with a blank panel, or substituted by a descriptive sentence. The reading time of the end state panel, which directly follows the manipulated bridging event panel, was compared across the conditions. The results showed that the blank panel and absent conditions led to the longest viewing times, suggesting high cognitive demands for generating inferences and retrieving missing information when reading pictorial stories. More importantly, viewing times in the text condition were longer than in the present condition, and the authors thus concluded that processing bridging information across texts and pictures requires additional cognitive effort. Based on these findings, the authors further suggested that there might be specific bases that differ between textual and pictorial presentations for initial processing. When a substitution happens, code-switching and recoding processes are involved, leading to additional mental effort. This conclusion contradicts those made by Cohn and Schilperoord (2022) and Manfredi et al. (2017), and further research is thus needed to clarify whether integrating text and pictures is indeed cognitively demanding.

Furthermore, a significant gap in the existing literature is that prior studies have only used images as story contexts, without examining the reverse scenario where text serves as the primary context. This raises the possibility that the observed “switching” costs could be attributed to the greater difficulty of processing verbal narratives compared to visual narratives, aligning with the widely held “visual ease” assumption (Coderre, 2020). Pictures tend to explicitly convey information, potentially requiring less cognitive resources. For instance, Larkin and Simon (1987) compared the computational efficiency of sentential versus diagrammatic presentations in solving mathematics and physics problems and found that diagrams are often superior to texts because they offer a straightforward presentation of information, thereby significantly speeding up the information search process. Consequently, further research is needed to explore these dynamics across both modalities.

Meanwhile, it would be interesting to investigate how individual differences, especially WM capacities, influence TPI. Indeed, Gyselinck et al. (2000) compared two groups of participants who read texts with or without illustrating pictures, respectively. Comprehension of the texts was tested using factual and inferential questions. The results demonstrated that the visuospatial WM is involved in the integration of texts and pictures. Participants with a higher visuospatial WM capacity were also better comprehenders of multimodal materials. However, such effects have not been confirmed in substitute instances, and more studies are still needed.

### Writing systems and reading comprehension

Another potential influencing factor in the area has been neglected, namely how the language, especially the writing system of the text, influences TPI. The rationale behind this query lies in the fact that reading acquisition involves learning how the writing system, which visually represents spoken language based on specific scripts and governing rules (Daniels & Bright, 1996) in a language functions. According to Perfetti and Dunlap (2008), this process involves decoding written symbols and mapping them onto linguistic representations. Such symbols and mapping relations can vary greatly across languages. In alphabetic languages such as English, letters are used to represent phonemes in spoken language (Pulgram, 1951); in logographic languages like Mandarin Chinese (henceforth, Chinese), by contrast, characters encode meaning and morphemes (Chen et al., 1996).

It is worthwhile to discuss the unique features of logographic Chinese texts. The most salient property of Chinese characters is that Chinese characters are box-shaped symbols that are visually complex (or relatively simple) (e.g.,  , /sen1/, *forest*;  , /mu4/, *tree*). Notably, many Chinese characters have similar visual forms (e.g.,  , /yi3/, *already*, and  , /ji3/, *self*;  , /qing2/, *sunny*;  , /jing1/, *eye*), highlighting the critical role of visual processing in Chinese character recognition (Chen et al., 1996).

Meanwhile, Chinese characters maintain a direct orthography-meaning relationship. Some characters are even pictograms or ideograms, which imitate the represented morpheme (e.g.,  , /ma3/, *horse*) or visualize the expressed concepts (e.g.,  , /xia4/, *down*). The majority of characters are compound characters with semantic radicals that indicate the meaning of the whole character (e.g.,  /huo3/ “fire” in  /shao1/ “to cook”).

Another crucial aspect of Chinese characters is their deep orthography. In other words, although each Chinese character is associated with a syllable, such a relationship is arbitrary and opaque. Moreover, given the limited number of available syllables in the language, the syllables are recycled to represent different meanings, resulting in a large number of homophones (Tan et al., 1996). These homophones can be very different in terms of orthography and meaning (e.g.,  , /wei3/, *great*;  , /wei3/, *tail*;  , /wei3/, *fake*; and  , /wei3/, *to wither*). As a result, phonological processing in Chinese typically occurs after whole-character recognition (Chen, 1995; Tan et al., 2005).

Given these logographic features, it is not surprising that visual skills are of great importance in Chinese character recognition (e.g., Huang and Hanley, 1995; Mcbride-Chang et al., 2005). This claim is supported by the priming effects observed when prime and target words share visual properties (e.g., Wang et al., 2010; Wang, 1998; Yang et al., 2023; Zhou and Marslen-Wilson, 1999). Chen and Kao (2002) found that visuospatial properties of Chinese characters serve as a perceptual basis for orthographic processing, with more properties leading to greater facilitation. By contrast, when processing alphabetic languages like English, phonology plays a central role in word recognition (Perfetti & Tan, 1998; Chen & Yuen, 1991). Studies have shown that processing written English relies more on phonological awareness and less on visual skills (Van Orden, 1987; Davis & Lupker, 2006). Such differences in writing systems have been shown to affect cognitive processes more generally. For instance, Zimmer and Fischer (2020) reported higher visuospatial WM capacities among Chinese readers compared to those of alphabetic languages.

Nonetheless, few studies have investigated how readers of different writing systems differ in picture processing. One such instance is Yum et al. (2011), where the authors recorded ERPs elicited by line drawing and English and Chinese words. The key finding was that readers of Chinese words, regardless of their first language, showed no word-picture differences in the early time window, whereas English words elicited a greater N170 effect. This finding suggests that alphabetic writing systems may involve more word-specific processing than logographic scripts in the early processing stage. Similarly, Yum and Law (2019) reported that characteristics of one’s native writing system can positively transfer to the processing of visual stimuli. Early experience with a visually complex orthography, such as Japanese Kanji, enhances visual recognition and semantic access when viewing line drawings, compared with alphabetic language speakers such as Korean.

Meanwhile, how integrating verbal and visual information may differ across writing systems has not been scrutinized. It is reasonable to hypothesize that logographic Chinese readers may exhibit an advantage in TPI. On the one hand, logographic script processing can be more picture-like (Yum et al., 2011); on the other hand, Chinese character recognition inherently involves a process of information integration: Chinese texts are, by definition, verbal presentation, but the orthography of the characters offers a huge amount of visual information. In this sense, Chinese readers might be more “trained” in visual processing and may already incorporate visual information into verbal processing. However, previous studies on TPI have only focused on Indo-European alphabetic languages, and cross-scriptal comparisons are highly needed.

## Current study

### Experiment overview, research questions, and hypotheses

**Fig. 1:**
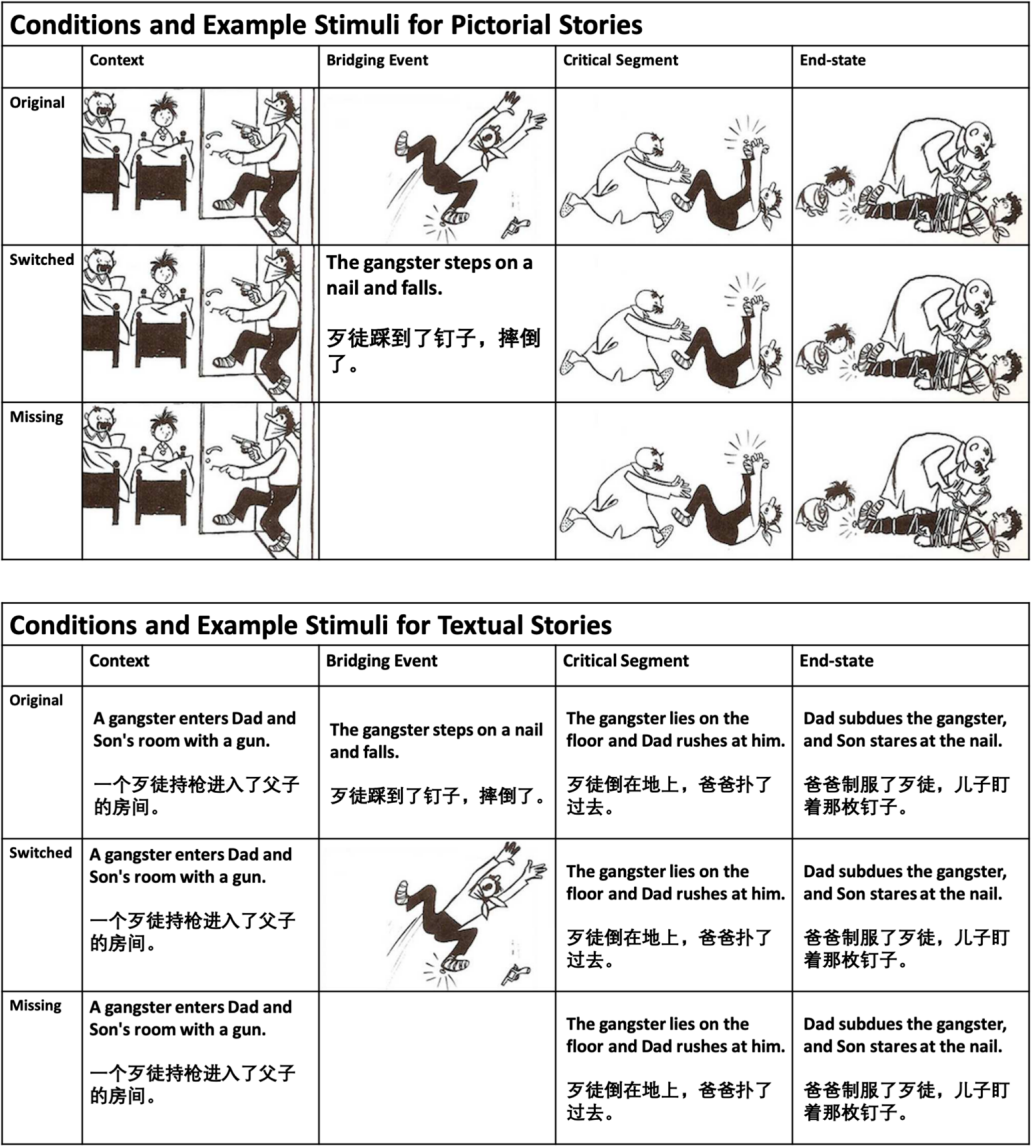
Conditions and example stimuli for the pictorial and textual stories. The textual stimuli were presented in English for the English L1 and L2 Groups, and in Chinese for the Chinese L1 and L2 Groups (Only One Language Shown per Group)

In the current study, we report a behavioral experiment investigating bridging inference generation in both verbal and visual narratives, as well as text-picture integration during bridging event processing. We employed a self-paced viewing/reading paradigm in which participants read pictorial or textual stories. Each story consisted of four segments, i.e., four panels or four sentences (cf. Figure 1). The stories always began with a context segment, followed by the bridging event. The bridging event was then manipulated to create three conditions in each story type:

1. *Original*, where all four segments of the stories were identical in modality. This condition serves as the baseline;
2. *Switched*, where the bridging event was replaced by a sentence in pictorial stories, or by a picture in textual stories. This condition aimed to investigate whether text-picture integration in substitution instances leads to increased cognitive effort;
3. *Missing*, where the bridging event was replaced by a blank panel. In this condition, bridging information was absent and thus a bridging inference had to be generated. We expected to replicate previous results in showing bridging inference is crucial to reading comprehension in both verbal and visual narratives.

We decided to manipulate the bridging event segment instead of the peak segment (Cohn, 2014b; Magliano et al., 2017), as the bridging event serves as a critical link in establishing causal relationships between the initiating event and the peak event. This approach thus allowed us to examine how participants reconstruct this missing connection and whether they can generate bridging inferences. While omitting the peak may create a more noticeable gap, its salience in the narrative structure could reduce the cognitive effort required to “fill in the blank.” By contrast, the bridging event forces participants to infer a subtler but crucial causal step, thus offering greater insight into their inferential abilities.

The third segment was the critical segment, as comprehension of this panel depended on bridging information or inference from the previous segment; thus, the reading time of this segment was directly affected by the manipulation. We recorded the viewing times or reading times of this segment, as well as the understanding rating of the stories, as the dependent variable, and then compared them across the three conditions within each story type. Finally, an end-state segment followed the critical segment to avoid any wrap-up effects.

Meanwhile, we investigated the effect of the writing system on the TPI process. To be more specific, we recruited L1 and L2 English and Chinese speakers and compared their comprehension times across the conditions. Given the differences in cognitive processing by readers of distinct writing systems, we anticipated differences in how these groups integrate text and pictures.

In addition, we explored how individual differences, such as WM capacity and visual language fluency, influence the *missing* costs and *switching* costs. These costs were calculated by subtracting the viewing or reading times of the critical segment in the original condition from those in the missing and switching conditions, respectively.

To summarize, we addressed the following research questions and proposed corresponding hypotheses:

#### Question 1

Can L1 and L2 English and Chinese speakers integrate bridging information across text and pictures?

#### Hypothesis 1

All groups should demonstrate successful TPI. If there is no difference between the original and switched conditions in comprehension ratings or the comprehension times of the critical segment, TPI likely requires no additional cognitive resources. However, if the switched condition yields longer comprehension times or lower ratings, this process may be cognitively demanding. The former outcome supports an integrated mental model encompassing both text and pictorial information (e.g., Cohn and Schilperoord, 2022), whereas the latter outcome supports models requiring separate mental representations for different modalities (e.g., Schnotz, 2014).

#### Question 2

Will bridging inference be generated when no bridging event is present in the pictorial and textual stories?

#### Hypothesis 2

It is expected that bridging inference is universal across modalities, and all groups of participants will be sensitive to the absent bridging event. However, we expect this inferential process to be cognitively demanding, evidenced by lower comprehension ratings and longer comprehension times for the critical segment in the missing condition.

#### Question 3

Do participants from different language backgrounds differ in their TPI?

#### Hypothesis 3

TPI is expected to be universal across languages. Hence, both English- and Chinese-speaking participants, including L2 learners, can integrate information across text and pictures. Nonetheless, given the distinct writing systems, L1 Chinese speakers may hold an advantage in this process, leading to smaller switch effects in this group.

#### Question 4

Do individual differences affect inferential generation and TPI?

**Table 1:** Demographic information and cognitive measures of the participants

	English L1	Chinese L1	English L2	Chinese L2
Gender (%)				
Male	29 (50.00%)	25 (36.23%)	16 (25.00%)	18 (28.57%)
Female	29 (50.00%)	44 (63.77%)	47 (73.44%)	41 (65.08%)
Mean age ***	37.46 (13.56)	36.55 (10.03)	31.41 (9.58)	29.75 (8.27)
Education level ***	4.48 (1.06)	4.91 (0.49)	5.05 (1.17)	5.11 (0.97)
SES ***	4.00 (1.51)	3.53 (0.99)	3.70 (1.47)	4.81 (1.63)
2-back score ***	0.85 (0.12)	0.82 (0.15)	0.91 (0.09)	0.88 (0.13)
Backward Corsi span **	4.62 (2.30)	5.42 (2.03)	5.97 (2.17)	5.14 (2.40)
VLFI score *	10.03 (8.59)	8.60 (6.69)	12.12 (8.47)	14.32 (10.13)
L2 proficiency	/	/	24.17 (3.14)	22.30 (7.92)

*Note.* The standard deviations (SDs) are reported in brackets, and the asterisks indicate that there are significant differences across participant groups. The 2-back score was used as an indicator of the participants’ verbal WM capacity. It may take any value between 0 and 1. Visuospatial WM capacity was measured as the backward Corsi span, ranging from 2 to 9. Familiarity with visual languages was determined by scores on the VLFI questionnaire, ranging from 1.5 to 52.5. Scores below 8 indicate low familiarity, and above 20 is high. Scores in between denote average familiarity

#### Hypothesis 4

Participants with higher WM capacity should exhibit less disruption in the no-bridging condition than those with lower WM capacity. Furthermore, the roles of verbal and visuospatial WM may differ: participants with higher visuospatial WM capacity may exhibit stronger effects in the pictorial condition, whereas those with higher verbal WM capacity may show stronger effects in the textual condition.

Additionally, participants with higher visual language fluency should show less disruption in the switching condition, as they are more adept at integrating text and pictures.

### Participants

Four groups of participants were recruited in the current study, namely 69 Chinese monolingual speakers, 63 English monolingual speakers, 63 advanced L2 Chinese speakers whose L1 was English, and 59 advanced L2 English speakers whose L1 was Chinese. Monetary compensation was provided to participants for their involvement in the study. All participants read the information sheet and digitally signed the consent forms before participating in the experiment. Ethics approval was obtained from the Faculty of Modern and Medieval Languages and Linguistics, University of Cambridge, before data collection.

Prior to the formal experiment, participants completed a demographic questionnaire and the VLFI questionnaire, a tool designed by Cohn (2014a) to measure participants’ experience and expertise in reading comics. The Chinese L1 group completed the task in Chinese, which was translated and adapted by the first author. The Chinese version of the questionnaire can be found in Appendix A. Their verbal and visuospatial WM capacities were also measured with the 2-back task and the backward corsi span task, respectively. A detailed description of the tasks can be found in Appendix B. To be noted, 76 English monolinguals completed the main experiment, but 18 of them (23.68%) refused to continue to the cognitive measures or to provide demographic information. Therefore, their data were only used for the text-picture integration analysis, but not for the individual difference measures.

The demographic information and the cognitive measures of the subjects are reported in Table 1. Interestingly, the L2 groups had a higher verbal WM capacity than the monolinguals, indicating a positive effect of learning an L2 on verbal WM. The English L2 groups also had a higher visuospatial WM capacity than the English monolinguals, suggesting that learning Chinese might boost the visuospatial WM capacity as well.

**Fig. 2:**
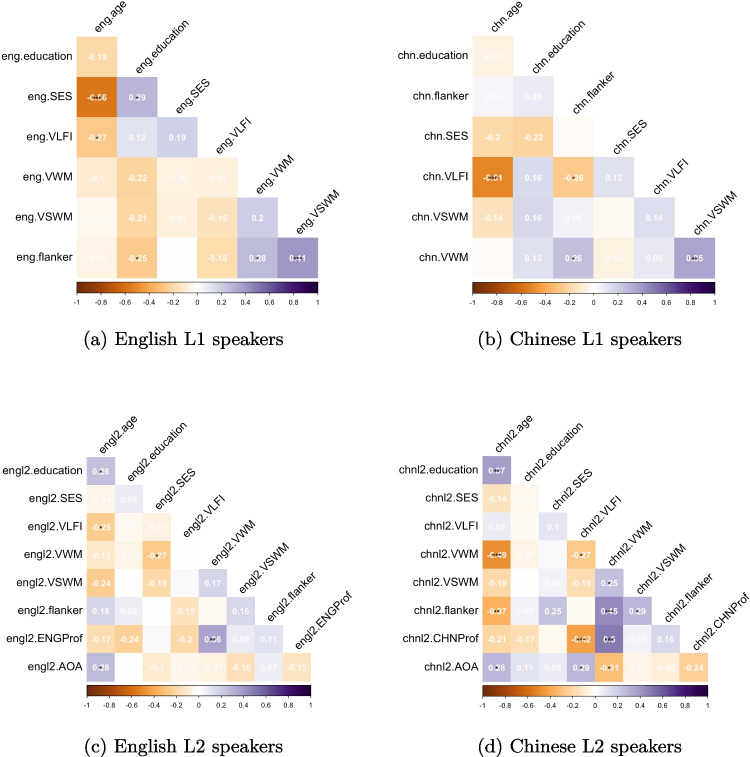
Correlation between the demographic, cognitive, and language measures of the four participants groups

The intercorrelations among the demographic and cognitive measures are reported in Fig. 2.

### Design and materials

The detailed experiment design and example stimuli are shown in Fig. 1. As there were six conditions, a 6x6 Latin Square design was adopted to create six versions in each language to ensure that each participant only encountered each story once. The Chinese L1 and L2 groups were tested in Chinese, while the English L1 and L2 groups received the experiment in English. The experiments were divided into two blocks, one with pictorial stories and one with textual stories. The presentation order of the two blocks was counterbalanced across participants.

The materials used in the current study were four-segment pictorial stories excerpted and adapted from *Father and Son* (German: *Vater und Sohn*; Plauen, 2018). Fifty-seven stories were selected and purposely edited so that no text was present in the pictures and a clear bridging event existed in each story. To create the textual stories, all pictorial stories were transcribed into English and Chinese by the first author. During transcription, the bridging event and critical sentences were constrained to 9–13 syllables in both languages. In each story, the syllable numbers in Chinese and English sentences differ by fewer than 2. We first ran a norming study to test whether the textual and pictorial stories were easy to understand, whether they were consistent with each other, and whether the bridging event in both texts and pictures convey clear bridging information. More details of the morning study are reported below.

#### Norming study

The selected stories were distributed across three lists using a Latin square design. Each list contained 19 textual stories, 19 pictorial stories, and 19 stories presented in both texts and pictures. 21 L1 speakers of each language who did not participate in the main experiment were recruited to evaluate one of the three lists. They rated their understanding of each story on a seven-point scale. For stories presented with both text and pictures, participants also assessed the congruence between the two modalities. For stories presented in either text or picture alone, participants rated the informativeness of the second segment (the bridging event) on a seven-point scale.

**Fig. 3:**
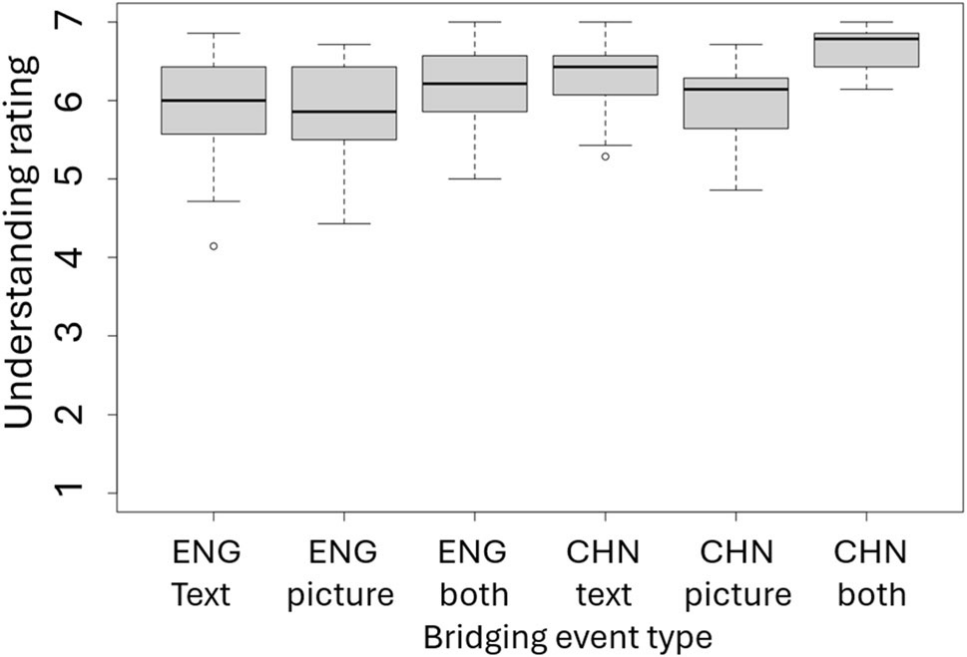
Norming test results: The mean understandability of the materials

**Fig. 4:**
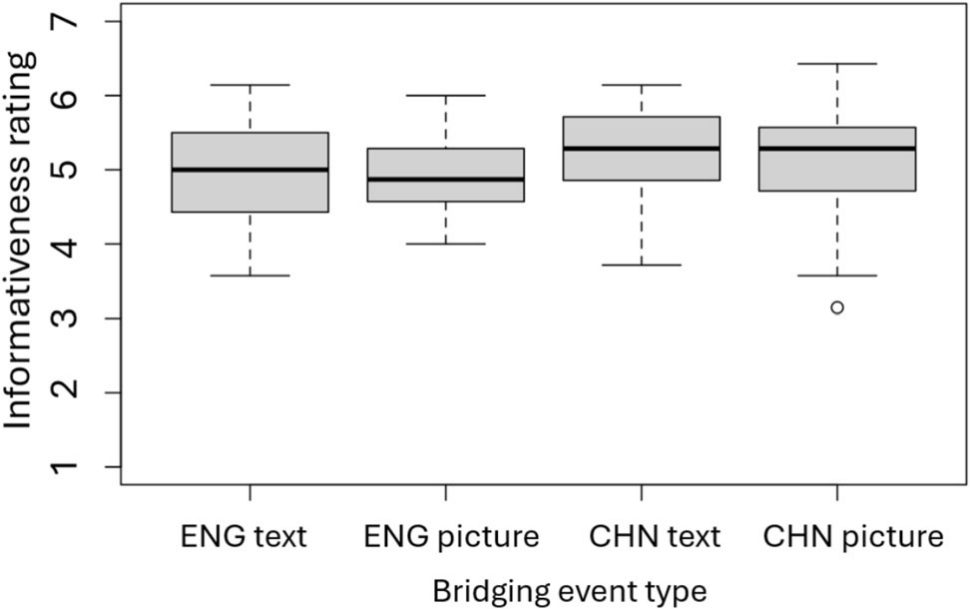
Norming test results: The average informativeness of the bridging information

**Fig. 5:**
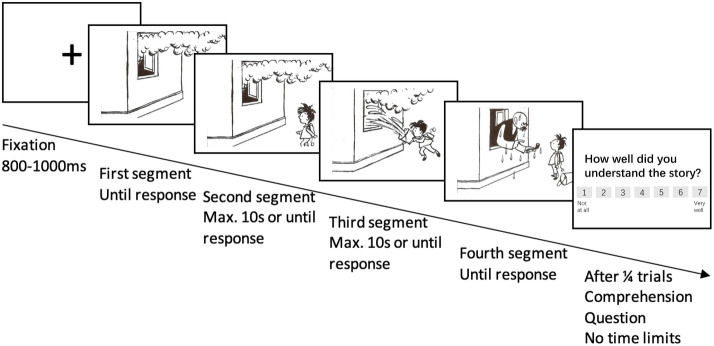
Procedure of the main experiment

The results demonstrated a high rating of congruence between pictures and texts (6.2 out of 7, cf. Figure 3). Statistical analyses revealed significant effects of presentation format on understanding in both English (F(2)= 5.85, *p* = .003) and Chinese (F(2)= 40.02, *p* < 0.001). Post hoc Tukey tests indicated that stories with both text and pictures were easier to comprehend compared to those presented in a single format, supporting the multimedia learning theory (Mayer, 2005). No differences were observed between the two presentation formats in both languages.

The informativeness of the bridging event segment is presented in Fig. 4. Paired *t* tests indicated no significant difference between the two presentation formats (for the English stories, *t*(47) = 0.32, *p* = .75; for the Chinese stories, *t*(47) = 0.55, *p* = .59).

### Procedure

The current experiment adopted the self-paced viewing time paradigm. The four segments in each story were sequentially presented and the presentation time was determined by the participants, who were asked to read and try to understand the stories as quickly as possible. Once they had understood each segment, i.e., they understood what the segments convey and how they relate to previous segments, they were asked to press the space bar to read the next segment. After reading each story, they were asked to rate their understanding of the whole story on a seven-point scale. A blank screen of 1300 ms was inserted between stories to avoid recency effects (cf. Fig. 5).

**Fig. 6:**
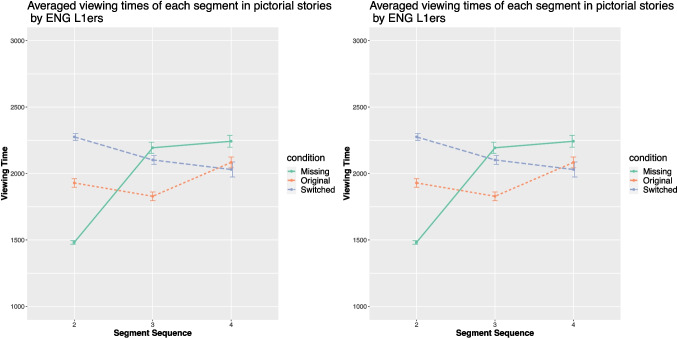
Averaged viewing times (in ms) of the English monolinguals in the three conditions

## Results

To answer the research question on whether TPI can be achieved during bridging information processing, the comprehension times of each segment, i.e., the time from stimuli presentation to key release, were analyzed. The data analysis was conducted in *R* (version 4.1.2; R Core Team, 2021). Reaction times that were below 300 ms or 3 SDs above the average were excluded from the data analysis (for English L1 speakers, *n* = 81 (2.91%); for Chinese L1 speakers, *n* = 162 (5.92%); for English L2 speakers, *n* = 147 (4.86%); for Chinese L2 speakers, *n* = 63 (2.05%)). The reaction times were then log-transformed to fit a normal distribution.

Firstly, to obtain an overall picture of the impact of Group and Condition on the comprehension times of the critical segment in both story types, linear mixed-effects models were built with the lme4 package (Bates et al., 2015) on the reading times of the critical segment in textual stories and the viewing times of the critical segment in pictorial stories, respectively. Condition (missing, original, switched) and Group (English L1, English L2, Chinese L1, Chinese L2) and their interactions were included as fixed effects, and random slopes for both Subjects and Stimuli were added as random effects. Type II ANOVA in the *car* package (Fox and Weisberg, 2019; version: 3.0.12) was employed to analyze the parameters in the models. The *effectsize* package was used to calculate eta-squared values which serve as the indicators of effect sizes for the ANOVA tests (Ben-Shachar et al., 2020), and the *emmeans* package was used for pairwise comparisons between group means (Lenth, 2023).

For pictorial stories, significant main effects of both Conditions ($\chi ^{2}$(2) = 42.34, *p* < .001, $\eta ^{2}$*p* = .66 (95% CI [0.42, 1.00])) and language group ($\chi ^{2}$(3) = 41.19, *p* < .001, $\eta ^{2}$*p* = .01 (95% CI [.00, 1.00])) were revealed. The interaction effect between Condition and Group was also significant (F(6) = 15.14, *p* < .02, $\eta ^{2}$*p* = .003 (95% CI [.00, 1.00]).

For textual stories, significant main effects of both conditions ($\chi ^{2}$(2) = 14.49, *p* $< .001$, $\eta ^{2}$*p* = .41 (95% CI [0.42, 1.00])) and language group ($\chi ^{2}$(3) = 377.57, *p* < .001, $\eta ^{2}$*p* = .07 (95% CI [.06, 1.00])) were revealed. The interaction effect between Condition and Group was not significant (F(6) = 15.14, *p* $=$ .70, $\eta ^{2}$*p* = .0007 (95% CI [.00, 1.00]).

### English L1 speakers

The averaged comprehension times of the bridging event, critical segment, and end-state segment in pictorial and textual stories in the three conditions by the English monolinguals are demonstrated in Fig. 6.

When reading pictorial stories, significant effects of Condition were observed on the viewing times of the critical panel ($\chi ^{2}$ (2,171) = 33.35, *p* < .001, $\eta ^{2}$*p* = .23 (95% CI [0.12, 1.00])). Post hoc analyses revealed that the viewing time of the critical panel was significantly shorter in the original condition than in the other two conditions (*p* < .001). No differences were demonstrated between the switched and missing conditions.

When reading textual stories, the mixed-effect ANOVA again revealed a significant effect of Condition on the reading time of the critical sentence ($\chi ^{2}$ (2, 171) = 6.82, *p* = .03, $\eta ^{2}$*p* = .06 (95% CI [0, 1])). The pairwise comparison of the reading time of the critical segment across the three conditions also only revealed a significantly longer reading time in the missing condition (*p* = .03).

### Chinese L1 speakers

**Fig. 7:**
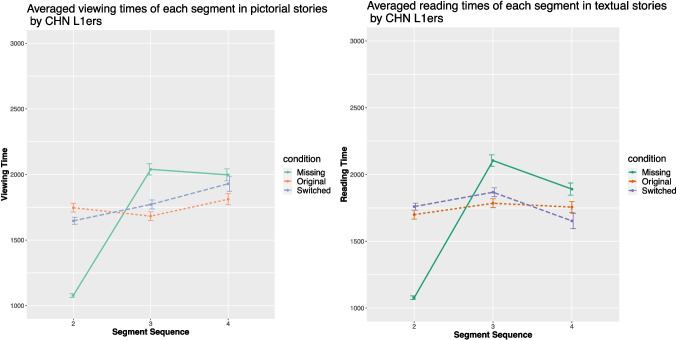
Averaged viewing times (in ms) of the Chinese monolinguals in the three conditions

Figure 7 presents the mean viewing times of each segment in both story types across the three conditions by the Chinese monolinguals. Mixed-effects ANOVAs revealed that Condition had a significant effect on the viewing time of the critical segment ($\chi ^{2}$ (2, 168) = 16.92, *p* < .001, $\eta ^{2}$*p* = .13 (95% CI [0.04, 1.00])). Post hoc Tukey HSD tests revealed a difference in the viewing times of the critical panel between the missing condition and the original condition (*p* < 0.05).

When reading the textual stories, the same effects were obtained ($\chi ^{2}$ (2, 168) = 14.28, *p* < .001, $\eta ^{2}$*p* = .11 (95% CI [0.03, 1.00])). The post hoc analyses revealed the same results as in the pictorial stories, i.e., the viewing time of the critical segment was longest in the missing condition, while there was no difference between the original and the switched conditions.

**Fig. 8:**
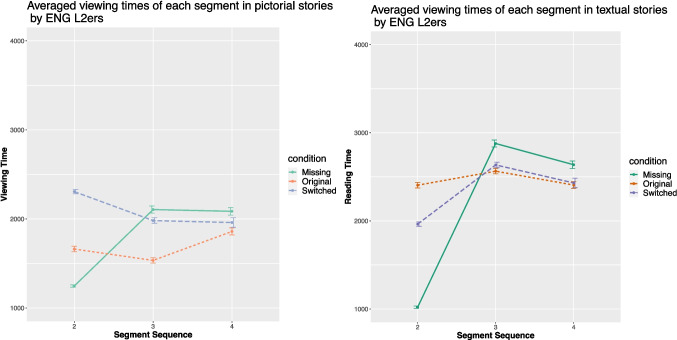
Averaged viewing times (in ms) of the English L2ers in the three conditions

### L2 groups

**Fig. 9:**
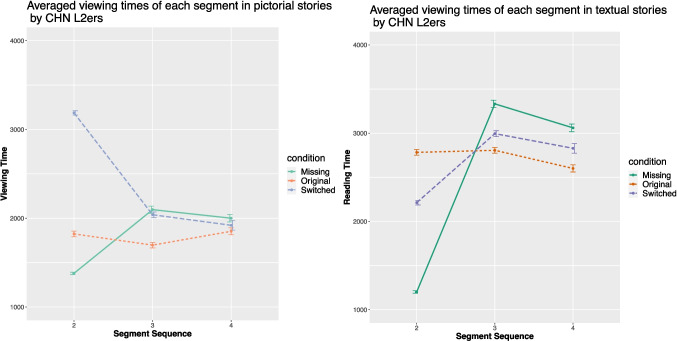
Averaged viewing times (in ms) of the Chinese L2ers in the three conditions

The English L2 comprehension times are plotted in Fig. 8 and the Chinese L2 data in Fig. 9. For both L2 groups, mixed-effect ANOVAs demonstrated Condition on the viewing time in pictorial stories of the critical segment (for the English L2ers, $\chi ^{2}$ (2, 189) = 100.73, *p* < .001, $\eta ^{2}$*p* = .44 (95% CI [0.34, 1.00]); for the Chinese L2ers, $\chi ^{2}$ (2, 186) = 46.47, *p* < .001, $\eta ^{2}$*p* = .27 (95% CI [0.16, 1.00])). The same patterns were obtained in the textual stories, i.e., significant effects on the reading time of the critical segment (for the English L2ers, $\chi ^{2}$ (2, 189) = 15.91, *p* < .001, $\eta ^{2}$*p* = .11 (95% CI [0.03, 1.00]); for the Chinese L2ers, $\chi ^{2}$ (2, 186) = 21.52, *p* < .001, $\eta ^{2}$*p* = .15 (95% CI [0.06, 1.00])).

**Fig. 10:**
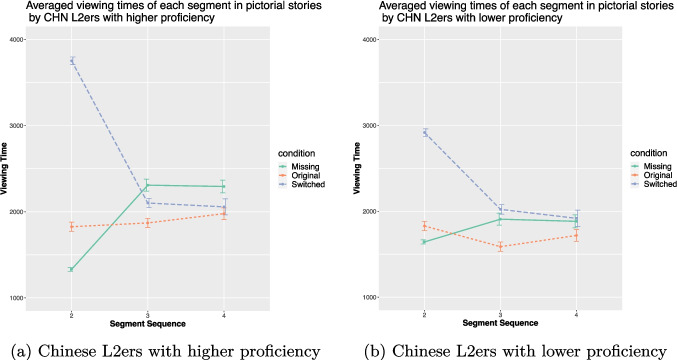
Averaged viewing times (in ms) of the pictorial stories in the three conditions by the Chinese L2ers divided by proficiency

For both L2 groups, the results from post hoc Tukey HSD tests mirrored those of the English L1 speakers. To be more specific, the viewing time of the critical segment in textual and pictorial stories differed. In pictorial stories, viewing time was significantly shorter in the original condition compared to the other two conditions (*p* < .001). In textual stories, the reading time of the critical sentence was significantly shorter in the original condition compared to the missing condition (*p* < .001), with no difference observed between the switched and original conditions.

**Fig. 11:**
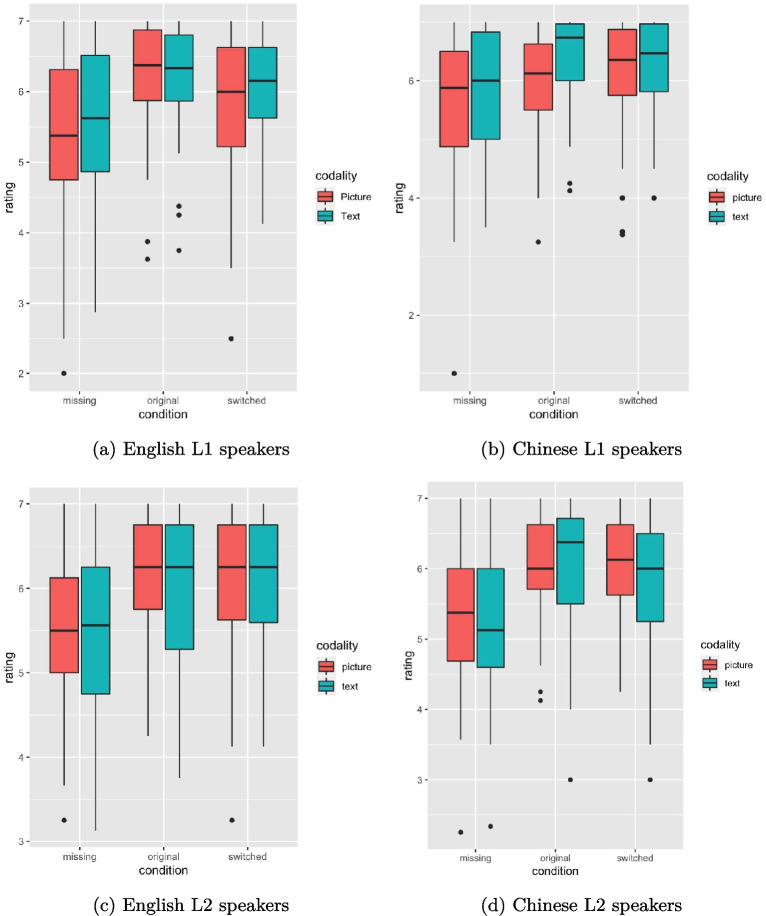
Understandability rating of the stories in each condition by each group

To test whether the high-proficient Chinese L2 speakers will perform more like the Chinese L1 speakers, the Chinese L2 group was further divided into two groups based on their proficiency levels (cf. Figure 10). The high-proficiency group (HP) comprised 21 Chinese L2 speakers who achieved a score above 27 on the Chinese proficiency test (M = 28.76, SD = 0.54), while the low-proficiency group (LP) included 21 participants with scores 27 or under (M = 12.71, SD = 6.46). When reading textual stories, mixed-effect ANOVAs revealed significant effects of Condition for both groups for the critical segment (for HP, $\chi ^{2}$ (2, 60) = 6.99, *p* = .03, $\eta ^{2}$*p* = .15 (95% CI [0, 1.00]); for LP, $\chi ^{2}$ (2, 60) = 6.76, *p* = .03, $\eta ^{2}$*p* = .15 (95% CI [0, 1.00])). Post hoc analysis indicated that both groups exhibited longer comprehension times for the critical segment in the missing condition compared to the other two conditions (*p* < .05). There were no differences between the missing and switched conditions.

When reading pictorial stories, significant effects of Condition were again observed for the critical segment (for HP, $\chi ^{2}$ (2, 60) = 14.82, *p* < .001, $\eta ^{2}$*p* = .27 (95% CI [0.08, 1.00]); for LP, $\chi ^{2}$ (2, 60) = 15.38, *p* < .001, $\eta ^{2}$*p* = . 28 (95% CI [0.08, 1.00])). However, post hoc analyses revealed different patterns between the two groups. The HP group required marginally more time in the missing condition than in the original condition (*p* = .05), while the LP group needed more time in both the missing and switched conditions compared to the original condition (*p* < .05 and *p* < .005, respectively).

### Understandability ratings

In addition to the viewing time data, the understandability ratings of the stories across these conditions yielded interesting findings. Kruskal–Wallis test revealed a significant effect of condition on the understandability ratings for all groups (for all groups, *p* $< .001$). Post hoc pairwise comparisons indicated that all groups rated the stories in the switched condition as equally understandable as the original stories, whereas stories lacking bridging information were consistently rated as significantly more difficult to comprehend (cf. Figure 11).

### Individual differences

To look at how individual differences interact with the conditions, linear regression models were built with the *lme4* package (Bates et al., 2015; version: 3.1.3). The switching costs (the subtraction of the comprehension times of the critical segment in the original condition from that in the switched condition) and the missing information costs (the subtraction of the reading time of the critical segment in the original condition from that in the missing condition) were entered as the dependent variables, respectively. The individual differences, namely verbal and visuospatial WM capacities, visual language fluency, and L2 proficiency where eligible, were entered as independent variables. Given the significant difference between the groups in education level, these two factors were also entered as independent variables.

The model on the performance of the English L1 speakers demonstrated that those with a higher verbal WM capacity were more likely to suffer from a larger picture-to-text switch cost and a larger text missing effect ($\beta$ = 5575.67, SE = 1884.46, *p* = .005; $\beta$ = 3980.33, SE = 1962.67, *p* = .048, respectively). The model also revealed significant effects of education level on text-to-picture switch effects and text missing effects ($\beta$ = 214.13, SE = 89.47, *p* = .02; $\beta$ = 576.89, SE = 220.64, *p* = .01, respectively), as well as SES on the picture missing effect ($\beta$ = -113.17, SE = 43.01, *p* = .01).

For the Chinese speakers, no significant effects of the individual difference on the switch costs in both directions were found. As for the missing effects, the subjects with a higher visuospatial WM capacity were less susceptible to the text-missing effect ($\beta$ = -88.07, SE = 39.45, *p* = .03).

As for the Chinese L2 speakers, the VLFI had a marginal negative effect on the picture-to-text switch effect ($\beta$ = -15.92, SE = 8.08, *p* = .05). The visuospatial WM, on the other hand, had a negative effect on the text-to-picture switch effect, the text missing effect, and the picture-missing effect ($\beta$ = -251.28, SE = 95.53, *p* = .01; $\beta$ = -142.71, SE = 64.40, *p* = .03; $\beta$ = -135.69, SE = 62.56, *p* = .03, respectively).

For the English L2 speakers, only a negative effect of the VLFI was observed on the picture-to-text switch effect ($\beta$ = -18.93, SE = 9.32, *p* = .047).

## Discussion

The current study investigated bridging inferential processing in both textual and pictorial stories, as well as the integration of text and pictures during the processing of bridging information. The influence of individual differences on these processes was also explored. To this end, L1 and L2 Chinese and English speakers were recruited to read and view pictorial and textual stories in which the bridging event was presented as either text, a picture, or a blank panel. Reading and viewing times for the segment following these manipulations were recorded and analyzed. Measures of WM capacity, visual language fluency, and, where applicable, L2 proficiency were also collected.

The findings revealed that TPI can be achieved during bridging information processing, and this process does not require extra cognitive resources. In contrast, generating bridging inferences is cognitively more demanding, and the absence of bridging information disrupts story comprehension. Notably, when an English – but not a Chinese – text replaced a picture, longer viewing times were observed at the subsequent picture, possibly due to the phonological decoding process. This effect was more pronounced when an L2 text was presented, particularly among participants with lower proficiency. These results are discussed in greater detail below.

### Bridging inferences are generated in both textual and pictorial stories

The first research question of the current study is, whether bridging inference can be generated when the bridging event is missing in both textual and pictorial stories. We thus compared the reading or viewing times in the missing conditions with those in the original condition. As predicted, missing conditions gave rise to prolonged comprehension times of the critical segment and lower ratings of the understandability of the stories, regardless of the story type. These results can be attributed to the fact that when the bridging event is missing, readers need to generate bridging inferences to fill the gaps between the given events. This finding joins the existing literature to suggest that inferential processing is universal across textual (e.g., Graesser et al., 1997; Magliano et al., 1993) and pictorial story comprehension (e.g., Cohn and Kutas, 2015a; Magliano and Zacks, 2011). Meanwhile, this finding is in line with previous studies that inferential generation is cognitively demanding (Fincher-Kiefer & D’Agostino, 2004; Magliano et al., 2016). Therefore, the absence of the bridging information leads to a distortion in reading comprehension, as reflected in the lower rating and longer comprehension times in the missing conditions compared with the original stories.

### TPI does NOT require extra cognitive resources

More importantly, the current study demonstrated that bridging information processing and integration across texts and pictures does not require additional cognitive resources. The presence of explicit bridging information, regardless of whether a switch was involved, facilitated reading comprehension. All groups rated the stories in the switched conditions as equally understandable as the original stories, regardless of the story modality. Moreover, for all groups, when a sentence is substituted with a picture in textual stories, no disruption in reading comprehension was caused. Likewise, when reading pictorial stories, the Chinese L1 speakers were also not affected by the bridging event conveyed in texts. These results indicate that the integration of text and pictorial information does not require extra cognitive resources, or at the very least, require fewer cognitive resources than inferential generation. In other words, bridging information across texts and pictures can be processed and integrated into a coherent mental representation during reading comprehension without additional effort.

This finding thus challenges the ITPC model (Schnotz, 2014), which posits that text and picture processing occur as separate models and require additional cognitive resources to align differing semantic structures during modality switching. Instead, our results support the view that a general comprehension skill underlies the interpretation of both verbal and nonverbal narratives (Gernsbacher et al., 1990). The ultimate goal of reading comprehension, i.e., the construction of a coherent situation model, is universal across textual and pictorial stories (cf. Zwaan and Radvansky, 1998). Likewise, the results align with the more recent multimodal parallel architecture framework (Cohn & Schilperoord, 2022), which emphasizes that texts and pictures are processed within the same mental architecture and a cohesive mental model is built from both inputs. Reading comprehension — whether of textual or pictorial stories — relies on incrementally integrating information into a coherent situation model, irrespective of modality. Crucially, this integration does not appear to demand extra cognitive resources or, at least, is less cognitively taxing than generating inferences.

### Writing systems differ in processing relative to picture processing

Nonetheless, the English L1 speakers demonstrated a slowdown effect when a picture was substituted with a sentence, which replicated the previous findings in Huff et al. (2020). However, this result should not be taken as evidence for the “visual ease” assumption (Coderre, 2020) or the claim that TPI is cognitively demanding. As discussed above, if these assumptions were true, we should have observed a similar effect in the Chinese L1 group. The interesting null results in this group invite us to find an alternative explanation for this contrast.

One of the possible explanations is that the prolonged reading time in English text processing might have been caused by the additional cognitive resources associated with alphabetic text processing compared with picture or logographic text processing. Anecdotally, processing Chinese texts does not appear to be more time-consuming than processing pictures (cf. Figure 7), whereas processing English texts appears to take longer than processing pictures (cf. Figure 6). Such contrast has also been reported in previous studies (e.g., Laubrock et al., 2018; Zhao et al., 2020), and again highlights the demands associated with English text processing.

The question then follows: Why are English texts harder to engage, while Chinese texts are not? Notably, a major difference between written English and Chinese lies in their writing systems. English is written in alphabetic letters, and English text processing requires the activation of phonological information; Chinese characters are logographic and hence call for visual skills in processing. Moreover, there might be a more direct path from the orthographic forms of the Chinese characters to meaning activation. As Laubrock et al. (2018) argued, “the amount of phonological and semantic information extracted from upcoming words differs between writing systems” (p. 242). When reading logographic characters, more semantic preview is offered, while alphabetic letters provide more phonological previews.

Such influence from writing systems has indeed been reported in previous studies. A meta-analysis by Li et al. (2021) demonstrated that the unique characteristics of writing systems lead to script-specific reading mechanisms. Differences in phonological encoding lead alphabetic and logographic readers to employ distinct pathways for accessing word meaning, and reading a logographic language involves more picture-like processes (Yum et al., 2011). Experience with a logographic language may also enhance the ability in visual information processing (Yum & Law, 2019). Therefore, the differences between English and Chinese L1 readers in the current study likely stem from these distinct processing pathways. While English readers rely on phonological decoding to access the semantics of the texts, Chinese readers retrieve meaning directly from the orthography - much like processing a picture. More studies are needed to validate this difference, and future models on TPI should incorporate such differences between writing systems.

### L2 texts pose challenges to reading comprehension

The results from the L2 groups provide further insight into the above discussion. Firstly, regarding inferential generation, the missing condition led to a longer reading time of the story, suggesting that the L2 speakers are actively retrieving the missing information and generating inferences. This is in line with previous studies which demonstrated that L2 speakers could generate inferences during reading comprehension to build global coherence, despite the greater difficulty in doing so (e.g., Foucart et al., 2016; Shimizu, 2015).

More importantly, both L2 groups performed similarly to the English L1 speakers in the switched condition. To be more specific, both L2 groups showed a slowdown effect when a text substituted a picture, but not the other way around. However, in the ad hoc analysis focusing on Chinese L2 speakers with high proficiency, no difference between the switched condition and the original condition in the text stories was observed (cf. Figure 10). This finding is comparable to the performance of the Chinese L1 speakers. Those with a lower Chinese proficiency, by contrast, found the texts significantly more difficult to process. For the less proficient L2 speakers, the logographic orthographic units are not directly associated with meanings, so the “shortcut” from the logographic text to meaning is blocked. In other words, phonological processing is obligatory for the less fluent Chinese readers.

### Individual differences have significant effects on TPI

The current study also explored how individual differences, including WM capacities and visual language fluency might influence the TPI process. Significant effects were indeed observed, which are further discussed below.

Firstly, visual language fluency had a positive correlation with the performance of both L2 speakers in the picture-to-text switch condition. This finding thus extends the claim by Cohn (2020) that the ability to comprehend pictorial stories is correlated with exposure and experience to these stories in situations where both texts and pictures are present. This effect is particularly significant on L2 speakers, as reading L2 texts poses greater challenges to the readers. In other words, not only does the ability to understand pictorial stories need to be “trained” through reading, but also the integration of information from texts and pictures, especially when the processing demand is high.

Secondly, only the Chinese L1 and L2 groups benefited from a higher visuospatial WM capacity. This pattern is in line with our hypothesis and previous studies suggesting that processing logographic writing systems relies more on visual processing skills and resources (e.g., Chen, 1996; Mcbride-Chang et al., 2005), while alphabetic text processing is not correlated with readers’ visuospatial WM capacity (Cole & Pickering, 2010). Notably, the L2 Chinese readers whose L1 is alphabetic also actively attended to the visual features when reading logographic characters, employing reading strategies distinct from their L1. This finding supports previous studies showing that L2 learners with higher visuospatial WM capacity learn visually enhanced characters more effectively (Kim et al., 2015).

Thirdly, the exploration of the verbal WM capacity yielded unexpected results. To be more specific, only the English L1 speakers were influenced by the verbal WM capacity when processing texts, but, against our hypothesis, readers with a higher verbal WM capacity tend to spend more time reading the stories with picture-to-text switch or generating inferences in textual stories. Nonetheless, this surprising result has also been reported in previous studies, especially when the cognitive load was high (e.g., Van Dyke and McElree, 2006; Nicenboim et al., 2016; Rai et al., 2011), and is consistent with the Attentional Control Theory (Eysenck et al., 2007). Readers with a higher WM capacity may be more strategic in reading and tend to prioritize comprehension over reading speed. Conversely, readers with a lower verbal WM capacity may struggle to fulfill tasks such as reading comprehension in the current study. Consequently, the failure in comprehension actually led to a null effect of the switched or missing information on those with a lower verbal WM capacity. On the other hand, those with a higher verbal WM capacity are engaged in more thorough comprehension and deeper parsing of the information, leading to prolonged comprehension times.

### Limitations

Despite the fruitful findings of this study, several limitations have to be noted. Firstly, only English and Chinese speakers were investigated in the current study. To validate the generalizability of the current results, future investigations should encompass a broader range of languages. For example, it would be interesting to explore how readers of Japanese, where both alphabetic and logographic scripts are used, perform in TPI. It would also be interesting to compare readers of English and German, where more transparency in sound-form mapping is offered.

Secondly, this study built on previous research on TPI (Huff et al., 2020; Magliano et al., 2017) and investigated the substitution of modalities in narratives. However, visual narratives often combine text and pictures within the same panel, and this is also a key focus of multimedia learning (Mayer, 2005; Schnotz, 2014). Future research should explore how text and pictures are integrated when presented simultaneously.

Finally, behavioral experiments may not be precise enough to reflect all the nuances in TPI. Therefore, further studies should be conducted with measurements that offer higher temporal resolution, such as ERPs and eye-tracking.

## Conclusion

The current study investigated TPI during bridging information processing by Chinese and English L1 and L2 speakers. The results demonstrated that bridging inferences can be generated during both textual and pictorial reading comprehension, which is a cognitively demanding process. TPI, however, does not require extra cognitive resources. Additionally, differences between alphabetic and logographic text processing are observed. Furthermore, the WM capacity and visual language fluency had overall positive correlations with the performance on bridging inference processing and TPI. Future research should employ more accurate measures such as ERPs to investigate TPI, and a wider range of languages should be examined.

## Figures and Tables

**Fig. 12:**
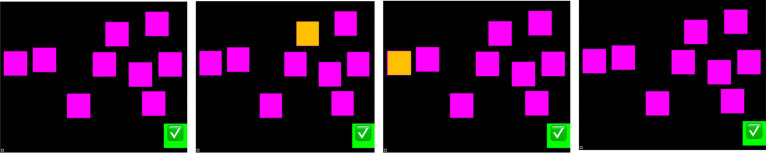
Illustration of the backward Corsi span task

## Data Availability

Data and materials have been made publicly available via the Open Science Framework and can be accessed at https://osf.io/evn5h/?view_only=0950b489089342d489f0a8e79fba9a93

## Appendix A The Chinese version of the VLFI questionnaire

1.

**Table 2:** Response options for question 1: Frequency of leisure activities involving text and pictures

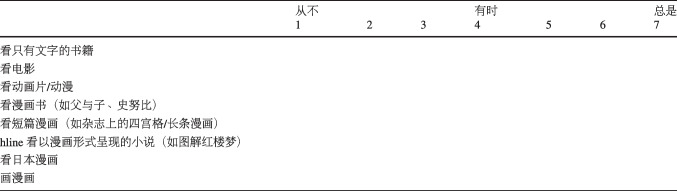

2.

**Table 3:** Response options for question 2: Frequency of leisure activities involving text and pictures before adulthood

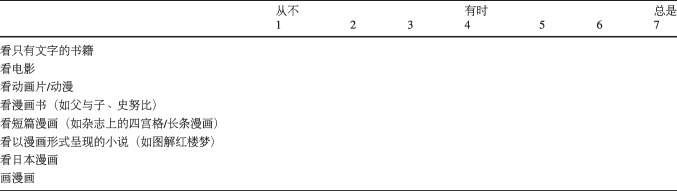

3.

**Table 4:** Response options for question 3: Self-ratings of the frequency, experience, and proficiency in reading comics

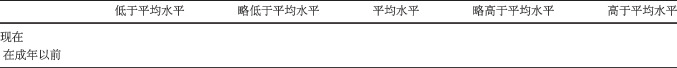

4.

**Table 5:** Response options for question 4: Self-ratings of frequency, experience, and proficiency in drawing

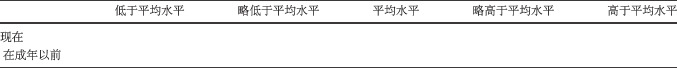

5.  __________

6.  __________

## Appendix B Individual difference measures

### Verbal working memory

The participants took a 2-back task to measure their verbal WM capacity (Kirchner, 1958). The participants saw a sequence of letters which appeared on the screen one by one. We used letters instead of digits or pictures to more explicitly activate the verbal component of the WM system. These letters were carefully chosen to make sure that they are also present in the Chinese Pinyin system^2^, and hence are familiar to the Chinese monolinguals and have a clear sound representation. Each letter was presented in white on a black background for 500 ms followed by a blank screen for 2500 ms. In each trial, participants were asked to determine whether the current letter matched the one presented two trials prior. If so, they needed to press the *m* key. If not, they were required to avoid pressing any keys. There were 60 trials in total, divided into four blocks, including one practice block.

To calculate the 2-back score, we adopted the composite A’ (A-prime) score developed by Zhang and Mueller (2005). It took into consideration both the hit rates (percentage of correctly identified matching letters) and false alarm rates (incorrect button presses). A higher A’ score indicates higher accuracy in the task and, consequently, greater verbal WM capacity.

### Visuospatial working memory

To assess their visuospatial WM capacity, the participants also took the backward Corsi span task (Corsi, 1973), where several blocks were presented on the screen. Each trial started with a beep sound, and then some of the blocks would change color (“highlighted”) one at a time (cf. Figure 12). The number of highlighted squares began at two and increased with each successful trial, with a maximum of nine highlighted blocks possible. Participants were asked to remember which blocks were highlighted and in what order. Following another beep, they were asked to click on the highlighted blocks in reverse other, i.e., starting with the last one highlighted. There was no time limit on the task. Upon completing this sequence, participants clicked on a green box at the bottom of the screen to mark the end of the trial. If participants made errors in two consecutive trials, the test terminated automatically. The backward Corsi span was determined by the longest sequence participants could accurately recall.

### Visual language fluency index

To assess the participants’ exposure to and familiarity with visual languages, the quantitative VLFI questionnaire developed by Cohn (2014a) was used. The questionnaire asks participants to indicate on a seven-point Likert scale (1 $=$ never and 7 $=$ always) how frequently they read all kinds of visual languages, including comic books, comic strips, graphic novels, and Japanese manga, and how often they watch visual presentations such as films or animations. They were also asked to rate their expertise in understanding these visual languages, as well as their drawing skills on a five-point Lickert scale (1 = below average and 5 = above average). The Chinese L1 speakers received the translated Chinese version.

The ratings of the participants were then calculated following the guidance of Cohn (2014a). A score above 20 is indicative of high visual language fluency, and a score below 8 suggests low exposure to and less familiarity with visual languages. Scores that lie between 10 and 19 imply an average level of visual language fluency. The full questionnaire can be downloaded here: https://www.visuallanguagelab.com/vlfi, and the Chinese translation can be found in Appendix A.

### L2 proficiency

Cloze tests were also conducted on L2 groups to measure their L2 proficiency. As proposed in Oller (1973), cloze tests evaluate not only language performance but language competence as well. They serve as a measure for all facets of language, involving lexical, syntactic, and even pragmatic knowledge (cf. Chung and Ahn, 2019; Yuan, 1993).

## Footnotes

1 The ITPC model offers a comprehensive explanation for text and picture comprehension in both auditory and visual modalities. However, in the current work, only the written modality, i.e., written texts and visual images, is concerned in the current work and described here
